# Low serum albumin, aspartate aminotransferase, and body mass are risk factors for frailty in elderly people with diabetes–a cross-sectional study

**DOI:** 10.1186/s12877-020-01601-z

**Published:** 2020-06-09

**Authors:** Ikumi Yanagita, Yuya Fujihara, Chikayo Iwaya, Yuichi Kitajima, Misuzu Tajima, Masanao Honda, Yuji Teruya, Hideko Asakawa, Tomoko Ito, Terumi Eda, Noriko Yamaguchi, Yumi Kayashima, Mihoko Yoshimoto, Mayumi Harada, Shoji Yoshimoto, Eiji Aida, Toshihiko Yanase, Hajime Nawata, Kazuo Muta

**Affiliations:** 1 Muta Hospital, 3-9-1 Hoshikuma, Sawara-ku, Fukuoka, 814-0163 Japan; 2 Fukuoka University, 7-45-1 Nanakuma, Jonan-ku, Fukuoka, 814-0180 Japan

**Keywords:** Frailty, Albumin, Transaminase, DHEA-S, Type 2 diabetes

## Abstract

**Background:**

Frailty is broadly characterized by vulnerability and decline in physical, mental and social activities and is more common in elderly patients with type 2 diabetes mellitus (T2DM). Frailty is closely associated with nutrition, muscle strength, inflammation, and hormones etc. In hormones, dehydroepiandrosterone sulfate (DHEA-S) and cortisol are suggested to be such candidates affecting frailty. Little investigation has been performed using a wider range of measures of frailty to clarify risk factors for frailty including the above two hormones.

**Methods:**

We performed a cross-sectional study to investigate the risk factors for frailty in elderly T2DM patients (*n* = 148; ≥65 years), using a broad assessment, the clinical frailty scale. We compared parameters between the non-frail and frail groups using the unpaired *t* and Mann-Whitney U tests. The Jonckheere-Therpstra test was used to identify relationships with the severity of frailty, and risk factors were identified using binary regression analysis.

**Results:**

Simple regression analysis identified a number of significant risk factors for frailty, including DHEAS < 70 μg/dL and cortisol/DHEA-S ratio ≥ 0.2. Multiple regression analysis showed that low albumin (< 4.0 g/dl) (odds ratio [OR] = 5.79, *p* < 0.001), low aspartate aminotransferase (AST) activity (< 25 IU/L) (OR = 4.34, *p* = 0.009), and low body mass (BM) (< 53 kg) (OR = 3.85, *p* = 0.012) were independent risk factors for frailty. A significant decrease in DHEA-S and a significant increase in the cortisol/DHEA-S ratio occurred alongside increases in the severity of frailty. DHEA-S concentration positively correlated with both serum albumin and BM.

**Conclusions:**

Hypoalbuminemia, low AST, and low BM are independent risk factors for frailty in elderly T2DM patients, strongly implying relative malnutrition in these frail patients. DHEA-S may be important for the maintenance of liver function and BM. A decrease in DHEA-S and an increase in the cortisol/DHEAS ratio may be involved in the mechanism of the effect of malnutrition in elderly T2DM patients.

## Background

In Japan, both men and women have long life expectancies. The aging population of Japan includes 10 million people with diabetes, and 50% of diabetes patients are elderly. Furthermore, it is predicted that the proportion of diabetes patients that are elderly will increase further in the future. Elderly people also frequently develop a geriatric syndrome that includes frailty. Frailty is a state of vulnerability and a consequence of cumulative decline in multiple physiologic systems over a lifespan, and is associated with a number of adverse outcomes, including falls, disability, hospitalization, care home admission, and mortality [1, 2]. Therefore, interventions are important for the achievement of optimal life expectancy. However, the risk factors for frailty have not been fully characterized.

The Clinical Frailty Scale (CFS) is thought to be the most suitable index for the quantification of frailty in elderly people because it includes physical, mental, and social scales [3]. We have previously shown, on the basis of a diagnosis of frailty made using the CFS, that 42% of 132 elderly patients with type 2 diabetes (T2DM) were frail and that aging and low circulating concentrations of albumin, high-density lipoprotein-cholesterol (HDL-C), systolic blood pressure (SBP), HbAlc, and total cholesterol were risk factors for frailty [4, 5]. Thus, the traditional risk factors for metabolic syndrome and/or cardiovascular disease in middle-aged people may shift from being deleterious to beneficial in old age. This shift, which is associated with malnutrition and chronic inflammation in elderly people, has been termed “reverse metabolism” [6, 7].

Sarcopenia is considered to be a risk factor for frailty in elderly people [8], and in our previous study of 108 T2DM patients of more than 65 years of age, we found that 35% had sarcopenia [9]. Serum DHEA-S increases adrenarche, peaks in a person’s 20s, and then decreases linearly with age to a concentration 10–20% of that of a young person [10, 11], suggesting that there might be an association between DHEA-S and the geriatric syndrome that includes sarcopenia and frailty. In our previous study, the elderly T2DM patients with sarcopenia had high serum cortisol concentrations and very low serum DHEA-S concentrations, presumably reflecting chronic stress. Furthermore, we showed that a cortisol/DHEA-S ratio of ≥0.2 is the strongest independent risk factor for sarcopenia [9].

In a cross-sectional study (mean age ± SD: 84.9 ± 9.6 years [12], 74.6 ± 7.7 years [13]) and a longitudinal study [14–16] (mean age ± SD at the start of study: 66.9 ± 2.2 years [14], 59 ± 11 years [15]) that used Fried’s method [2], which only involves assessment of the physical characteristics of the frailty, it was found that elderly people with relatively high DHEA-S have a relatively low risk of frailty. It has also been reported that elderly people classified as frail on the basis of the Fried method have high cortisol/DHEA-S ratios [14]. However, a broader assessment of frailty, like the CFS, has not been used to assess the roles of cortisol and DHEA-S in the frail elderly.

In our previous study of frailty in elderly T2DM patients [4], the roles of cortisol and DHEA-S were not investigated. Therefore, we performed the present study to determine the significance of these hormones for frailty in elderly T2DM patients, using the CFS. In addition, we aimed to re-evaluate the risk factors for frailty in elderly T2DM patients.

## Methods

### Participants

We retrospectively reviewed the data from 148 consecutive elderly T2DM patients older than 65 years (63 men and 85 women; 65–95 years). They were outpatients or hospitalized at Muta Hospital between October 2016 and September 2017. The exclusion criteria of the patients were described in our previous study [9]. The diagnosis of T2DM was based on the criteria by the Japan Diabetes Society [17] or a history of administering insulin or oral hypoglycemic agents. All data were obtained from the patients’ medical records as previously described [9]. This study was approved by the Institutional Ethics Committee (29–0001, May 15, 2017) and registered with UMIN (number 000031357).

### Hematology and hormone measurements

Blood sample collection and the measurements of biochemical markers in the blood were performed as previously described [9]. We also collected height, body mass (BM), and body mass index (BMI) data. BMI was calculated as the BM in kilograms divided by the height in meters, squared. SBP and diastolic blood pressure (DBP) were measured using a mercury sphygmomanometer at rest and in a sitting position. Measurements of serum levels of cortisol and DHEA-S were performed as previously described [9] using respective assay kits [18, 19]. The information on the detection limits and the assay variance for cortisol and DHEA-S was previously shown. The cortisol (μg/dL)/ DHEA-S (μg/dL) ratio was then calculated.

### Evaluation of frailty

The CFS containing nine grades [3] was used to evaluate frailty, as previously described [4, 9]. Briefly, patients with CFS grades 1 to 4 (1, very fit; 2, well; 3, managing; and 4, vulnerable) were defined as not being frail, whereas patients with CFS grades 5 to 9 (5, mildly frail; 6, moderately frail; 7, severely frail; 8, very severely frail; and 9, terminally ill) were defined as frail.

### Evaluation of sarcopenia

Sarcopenia was examined as previously described [9]. As recommended by the European Working Group on Sarcopenia in Older People (EWGSOP) [20] and Asian Working Group for Sarcopenia [21], three components, including muscular strength, physical function and muscle mass, were assessed using handgrip strength, walking speed and bioelectrical impedance, respectively. The severity of sarcopenia was evaluated using the criteria of the EWGSOP [20] as follows: pre-sarcopenia (a loss of muscle mass alone), sarcopenia (a loss of muscle mass plus a reduction in muscular strength or physical ability) and severe sarcopenia (a reduction in all three components). Participants with either sarcopenia or severe sarcopenia were considered to have “sarcopenia” for the purposes of the present study.

### Statistical analysis

Comparison of the non-frail and frail groups were made using the unpaired *t*-test or the Mann-Whitney U test according to the data distribution (with or without normality defined by the Shapiro-Wilk test). The Jonckheere-Therpstra test was used to determine the relationships of other parameters with the severity of frailty. The risk factors for frailty were identified using binary regression analysis, odds ratios (ORs), simple regression analysis, and multiple binary regression analysis. Receiver operating characteristic (ROC) analysis was performed and appropriate cut-off values were estimated for each risk factor. Fisher’s exact test was performed to compare the prevalence of sarcopenia between frail and non-frail participants. A *p*-value < 0.05 was considered significant.

## Results

Of the 148 elderly T2DM patients, 57 were frail (38.5%; 20 men and 37 women; CFS 5–7) and 91 (43 men and 48 women; CFS 1–4) were not frail. The CFS grades of the participants ranged from 1 to 7, with 18 having CFS1, 17 having CFS2, 27 having CFS3, 29 having CFS4, 15 having CFS5, 28 having CFS6, and 14 having CFS7.

Table 1 shows a comparison of the characteristics of the frail and non-frail elderly T2DM patients. Compared with the non-frail group, the frail group was significantly older (*p* < 0.001) and had lower BM (*p* < 0.001), BMI (*p* = 0.029), SBP (*p* = 0.013), DBP (*p* = 0.047), RBC (*p* = 0.005), Hb (*p* = 0.001), albumin (Alb) (*p* < 0.001), AST (*p* = 0.047), ALT (*p* = 0.017), eGFR (*p* = 0.007), HDL-C (*p* = 0.002), and Calcium (*p* = 0.011). Furthermore, compared with the non-frail group, the frail group had a significantly lower serum concentration of DHEA-S (*p* = 0.002) and significantly higher serum cortisol concentration (*p* = 0.022). As a result, the cortisol/DHEA-S ratio was significantly higher in the frail group than the non-frail group (*p* < 0.001).

**Table 1:** Characteristics of the elderly patients with diabetes, categorized according to frailty status

	All cases *N* = 148	Non Frailty *N* = 91	Frailty^a^ *N* = 57	*P* values
Age, years	76.9 ± 7.4	75.1 ± 6.8	79.8 ± 7.5	< 0.001 ^1)^
Male, n (%)	63 (42.6)	43 (47.3)	20 (35.1)	0.173 ^2)^
Body mass, kg	57.4 ± 11.1	61.8 ± 9.6	49.1 ± 8.5	< 0.001 ^1)^
BMI, kg/m^2^	23.2 ± 3.7	23.7 ± 3.5	22.3 ± 3.8	0.029 ^1)^
SBP, mmHg	135.5 ± 19.0	138.5 ± 18.8	130.6 ± 18.4	0.013 ^1)^
DBP, mmHg	72.4 ± 12.1	74.0 ± 12.2	69.9 ± 11.6	0.047 ^1)^
RBC, × 10^4^/μL	413 ± 58	424 ± 55	396 ± 59	0.005 ^1)^
Hemoglobin, g/dL	12.7 ± 1.7	13.1 ± 1.7	12.1 ± 1.6	0.001 ^1)^
Albumin, g/dL	3.95 ± 0.51	4.15 ± 0.32	3.63 ± 0.60	< 0.001 ^1)^
AST, IU/L	25.3 ± 10.7	26.7 ± 10.9	23.1 ± 10.3	0.047 ^1)^
ALT, IU/L	16.5 [12.0–25.3]	19.0 [13.0–27.0]	15.0 [10.0–22.0]	0.017 ^3)^
HbA1_c_, %	6.92 ± 0.84	6.99 ± 0.79	6.80 ± 0.90	0.171 ^1)^
S-Creatinine, mg/dL	0.70 [0.60–1.00]	0.70 [0.60–0.90]	0.80 [0.60–1.10]	0.125 ^3)^
eGFR, mL/min/1.73 m^2^	59.5 [40.0–73.4]	65.4 [52.3–74.4]	54.9 [38.4–69.1]	0.007 ^3)^
Uric acid, mg/dL	5.16 ± 1.43	5.06 ± 1.33	5.33 ± 1.57	0.265 ^1)^
Triglycerides, mg/dL	140 ± 77	139 ± 74	141 ± 82	0.911 ^1)^
LDL-C, mg/dL	101 ± 31	102 ± 31	99 ± 31	0.623 ^1)^
HDL-C, mg/dL	53.2 ± 14.3	56.0 ± 14.3	48.6 ± 13.4	0.002 ^1)^
Calcium, mg/dL	9.35 ± 0.49	9.27 ± 0.36	9.48 ± 0.63	0.011 ^1)^
DHEA-S, μg/dL	57.5 [39.0–89.0]	66.0 [44.5–106.0]	48.0 [33.0–68.0]	0.002 ^3)^
Cortisol, μg/dL	9.50 ± 2.79	9.09 ± 2.77	10.16 ± 2.71	0.022 ^1)^
Ratio Cortisol/ DHEA-S	0.16 [0.10–0.25]	0.13 [0.09–0.22]	0.20 [0.14–0.34]	< 0.001 ^3)^
Anti-HT drug use, n (%)	87 (58.8)	51 (56.0)	36 (63.2)	0.493 ^2)^
Anti-DL drug use, n (%)	72 (48.6)	44 (48.4)	28 (49.1)	0.999 ^2)^

Data are expressed as means ± SD or medians [25–75% values] or numbers (%)

^a^Frailty was defined using the clinical frailty score (≥5). *P* values were determined using^1)^ the unpaired *t*-test,^2)^ Fisher’s exact test^3)^ Mann-Whitney test. *BMI* body mass index, *SBP* systolic blood pressure, *DBP* diastolic blood pressure, *eGFR* estimated glomerular filtration rate, *LDL-C* low-density lipoprotein-cholesterol, *HDL-C* high-density lipoprotein-cholesterol, *DHEA-S* dehydroepiandrosterone sulfate, *HT* hypertension, *DL* dyslipidemia

To clarify the risk factors for frailty, simple regression analysis and multiple regression analysis using binary logistic regression were performed and ORs were calculated (Table 2). Simple regression analysis revealed that the significant risk factors for frailty were age ≥ 75 years (*p* = 0.005), BM < 53 kg (*p* = 0.002), RBC < 400 × 10 [4]/μL (*p* = 0.037), Hb < 13 g/dL (*p* = 0.003), Alb < 4.0 g/dL (*p* < 0.001), AST activity < 25 IU/L (*p* = 0.016), eGFR < 60 ml/min/1.73 m^2^ (*p* = 0.002), HDL-C < 40 mg/dL (*p* = 0.005), DHEA-S < 70 μg/dL (*p* = 0.007), and cortisol/DHEAS ratio ≥ 0.2 (*p* = 0.008). Multiple regression analysis showed that low Alb (< 4.0 g/dL) (OR = 5.79, *p* < 0.001), low AST activity (< 25 IU/L) (OR = 4.34, *p* = 0.009), and low BM (< 53 kg) (OR = 3.85, *p* = 0.012) were independent risk factors for frailty.

**Table 2:** Risk factors for frailty, determined using binary logistic regression analysis

Variables	Before adjustment		After adjustment	
	OR (95%CI)	*P* values	OR (95%CI)	*P* values
Age **≥** 75 years	2.74 (1.35–5.56)	0.005	0.97 (0.36–2.63)	0.956
Body mass < 53 kg^b^	3.03 (1.52–6.05)	0.002	3.85 (1.35–10.99)	0.012
SBP **≥**135 mmHg	0.69 (0.35–1.34)	0.270	0.56 (0.23–1.32)	0.184
RBC < 400 × 10^4^/μL	2.06 (1.04–4.08)	0.037	0.28 (0.08–1.03)	0.055
Hemoglobin < 13 g/dL^a^	2.87 (1.42–5.79)	0.003	2.72 (0.76–9.67)	0.122
Albumin < 4.0 g/dL^a^	6.50 (3.10–13.59)	< 0.001	5.79 (2.20–15.26)	< 0.001
AST < 25 IU/L^a^	2.40 (1.18–4.88)	0.016	4.34 (1.43–13.17)	0.009
ALT < 22 IU/L^a^	1.46 (0.71–2.99)	0.304	0.38 (0.12–1.27)	0.188
eGFR < 60 mL/min/1.73 m^2^	5.06 (1.83–13.98)	0.002	2.92 (0.83–10.27)	0.094
HDL-C < 40 mg/dL	3.44 (1.45–8.20)	0.005	0.97 (0.86–8.83)	0.089
Calcium **≥**9.4 mg/dL^a^	1.25 (0.64–2.45)	0.515	1.00 (0.41–2.45)	0.996
DHEA-S < 70 μg/dL^a^	2.78 (1.33–5.71)	0.007	2.19 (0.91–5.22)	0.081
Cortisol **≥**9.5 μg/dL^a^	1.75 (0.90–3.42)	0.100	1.75 (0.90–3.42)	0.100
Ratio Cortisol / DHEA-S **≥** 0.2	2.55 (1.27–5.10)	0.008	Not apply	

The chi-square value was 9.917 for the Hosmer-Lemeshow test (*p* = 0.271). ^a^The mean value was used. ^b^The median value was used. *OR* odds ratio, *CI* confidence interval, *eGFR* estimated glomerular filtration rate, *HDL-C* high-density lipoprotein-cholesterol, *DHEA-S* dehydroepiandrosterone sulfate

Next, ROC analysis was performed to determine appropriate cut-off values for the prediction of frailty (Table 3). The cut-off values calculated were 4.0 g/dL for Alb, 0.15 for cortisol/DHEA-S, 57 μg/dL for DHEA-S, 53 kg for BM, and 20 IU/L for AST. The AUC was highest for Alb (0.787), followed by cortisol/DHEA-S ratio (0.697), DHEA-S (0.653), BM (0.645), and AST (0.634).

**Table 3:** AUC for each predictor, determined using ROCs

Predictors	Cut-off values	AUC (95%CI)	*P* values
Body mass	53 kg	0.645 (0.549–0.741)	0.003
Albumin	4.0 g/dL	0.787 (0.710–0.864)	< 0.001
AST	20 IU/L	0.634 (0.541–0.728)	0.006
Ratio Cortisol / DHEA-S	0.15	0.697 (0.611–0.782)	< 0.001
DHEA-S	57 μg/dL	0.653 (0.584–0.741)	0.002

*AUC* area under the curve, *ROC* receiver-operating characteristic, *CI* confidence interval, *DHEA-S* dehydroepiandrosterone sulfate

**Fig. 1:**
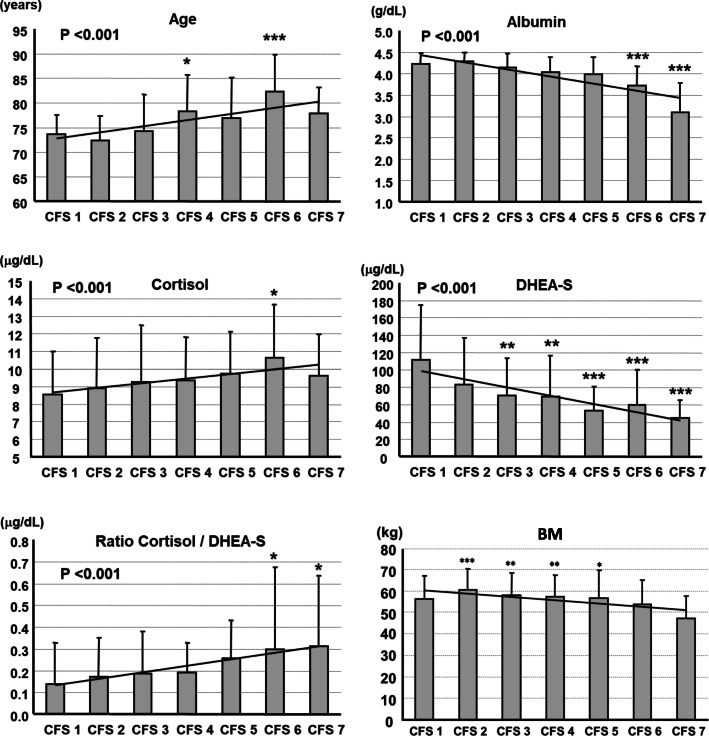
Predictive variables, classified using the Clinical Frailty Scale (CFS). Data are means + standard deviation (SD). *P* values for linear regressions were determined using the Jonckheere-Terpstra test. * *p* < 0.05, ** *p* < 0.01, *** *p* < 0.001 vs. CFS1, determined by multiple comparison using Fisher’s LSD test, following analysis of variance (ANOVA). For body mass, * *p* < 0.05, ** *p* < 0.01, *** *p* < 0.001 vs. CFS7, determined using Fisher’s LSD multiple comparison method, following ANOVA (*p* = 0.025)

The relationships between these predictive variables and the CFS are shown in Fig. 1. Significant reductions in Alb (*p* < 0.001) and DHEA-S (*p* < 0.001); and increases in age (*p* < 0.001), cortisol (*p* < 0.001), and cortisol/DHEA-S ratio (*p <* 0.001) occurred alongside an increase in the severity of frailty. In addition, BM was significantly decreased with an increase in the severity of CFS (Fig. 1).

**Fig. 2:**
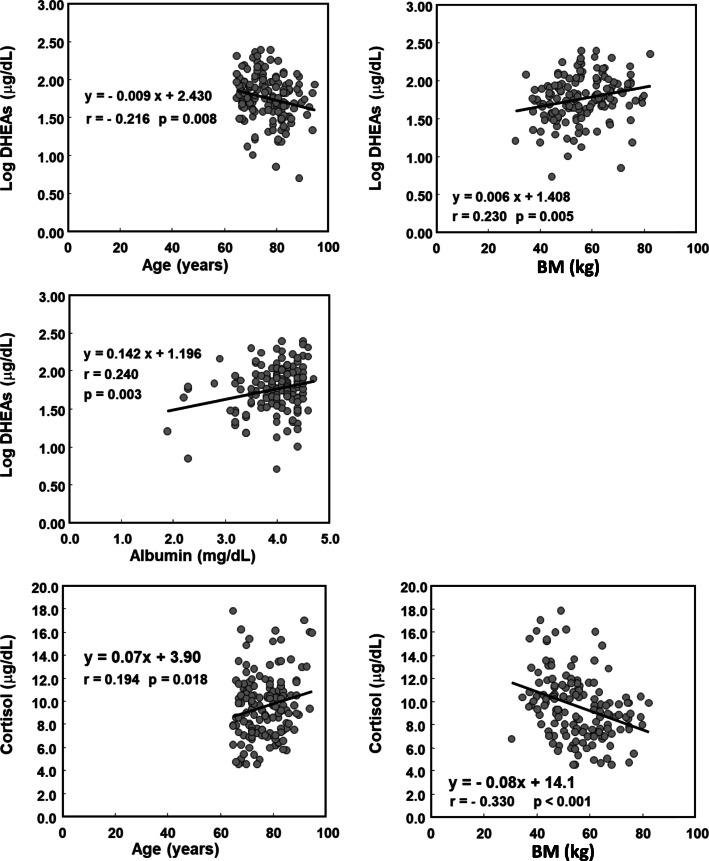
Relationships of log DHEA-S and cortisol concentrations with patient characteristics

We also analyzed the relationships of serum log DHEA-S and cortisol concentrations with age, serum Alb and BM (Fig. 2). Serum log DHEA-S significantly decreased (*r* = − 0.216, *p* = 0.008) and serum cortisol concentration significantly increased (*r* = 0.194, *p* = 0.018) with age. Serum Alb concentration significantly positively correlated with serum DHEA-S (*r* = 0.240, *p* = 0.003), but did not correlate with serum cortisol concentration (data not shown). BM significantly positively correlated with serum log DHEA-S (*r* = 0.230, *p* = 0.005) and significantly negatively correlated with serum cortisol (*r* = − 0.330, *p* < 0.001).

In the present study, of the 148 participants, 91 of whom were frail (CFS1–4) and 57 who were not (CFS 5–7), it was possible to evaluate whether 108 participants (32 of whom were frail and 76 were not) had sarcopenia because handgrip strength, walking speed, and bioelectrical impedance data were available. Fisher’s exact test revealed that the frail participants had a significantly higher prevalence of sarcopenia (50.0%, 16/32) than non-frail participants (28.9%; 22/76) (*p* = 0.048).

## Discussion

In the present study, we have identified independent risk factors for frailty, defined using the CFS, in 148 elderly T2DM patients, using multiple logistic regression analysis. These were serum Alb < 4.0 g/dl (*p* < 0.001), AST activity < 25 IU/L (*p* = 0.009), and BM < 53 kg (*p* = 0.012). In our previous study of 132 elderly T2DM patients, multiple regression analysis revealed that advanced age, and low albumin, HDL-C, SBP, HbA1c, and BM were risk factors for frailty, quantified using CFS, of which albumin was the most potent [4].

Low albumin was also identified to be the most potent risk factor for frailty in the present study. Therefore, we recommended that the serum albumin concentration of elderly people is maintained at ≥4.0 g/dL to prevent frailty. In Japan, protein intake is low in the elderly, which is likely to cause frailty. Furthermore, mortality in hospitalized patients is associated with hypoalbuminemia, and hypoalbuminemia has been shown to increase mortality [22].

In the present study, low Alb concentration and BM were shown to be independent risk factors for frailty, as shown previously, but HDL-C, SBP, and HbA1c were not. This is probably because the participants and sample size differed from those in the previous study, even though there was an overlap in the participants in each study (the number were 74 of 148 cases). However, although the list of risk factors differed between the studies, the common factors suggest that malnutrition or a related condition may be the most important cause of frailty in elderly T2DM patients. Malnutrition may be a constitutional syndrome or may be the result of strict dietary control imposed by the individual or their doctor. However, a number of endocrine factors may also be involved in malnutrition [5], as discussed below.

Interestingly, in the present study, the ALT and AST activities were significantly lower in the frail group than in the non-frail group, and AST < 25 IU/L was shown to be an independent risk factor for frailty. Recently, low serum ALT activity has been shown to be associated with aging [23], higher prevalence of frailty [23, 24] and sarcopenia [24] and to be a predictor of their subsequent higher mortality [23–25] in the elderly. Thus, ALT has been suggested to be a useful biomarker for aging and frailty.

However, there is no explanation for these associations. Low serum ALT and AST activities occur secondary to low serum concentrations of vitamin B6, because AST, and especially ALT, require vitamin B6 as a cofactor [26]. Furthermore, vitamin B6 deficiency has been shown to be associated with low activity and low serum albumin in elderly nursing home residents [27]. Thus, one plausible explanation for the low transaminase activities in frail T2DM patients may be relative vitamin B6 deficiency, resulting from malnutrition.

Although serum cortisol, DHEA-S and cortisol/DHEA-S ratio were not found to be independent risk factors in the multiple regression analysis, there is evidence that they contribute to frailty in elderly T2DM patients. In the present study, while log serum DHEA-S decreased significantly with age, even in the range of 65–95 years old, serum cortisol increased significantly. Serum DHEAS < 70 μg/dL was found to be a risk factor for frailty in simple regression analysis, but no significant relationship was identified between serum cortisol and frailty. Serum cortisol significantly increased, while serum DHEA-S significantly decreased with increasing CFS, between grades 1 and 7. As a result, a cortisol/DHEAS ratio ≥ 0.2 was a significant risk factor for frailty in simple regression analysis. As for elderly T2DM patients with sarcopenia [9], frail T2DM patients are thought to be under stress. Whereas cortisol is as catabolic hormone, DHEA-S is anabolic and antagonizes cortisol. Therefore, the relative cortisol overactivity may disrupt homeostasis, resulting in frailty and sarcopenia.

Interestingly, serum albumin concentration positively correlated with serum DHEA-S concentration (*r* = 0.240), suggesting that DHEA-S may have an anabolic effect in the liver. DHEA has also been suggested to reduce urinary albumin excretion in the kidney [28], which may also contribute to the maintenance of the serum albumin concentration. A protective action of DHEA-S in the liver has been also suggested by other studies: the serum DHEA-S concentration is low in non-alcoholic fatty liver disease (NAFLD) [29, 30] and DHEA reduces liver fat deposition in obese (*fa/fa*) Zucker rats [31]. The underlying molecular mechanism for the protective effect of DHEA-S in liver may involve the induction of CYP4A, which removes harmful substances. Alternatively, the dihydrotestosterone, ΔAdiol, and 3b-Adiol generated from DHEA-S may promote miR-21 production, leading to the proliferation of hepatocytes [32]. Therefore, it may be that the low serum DHEA-S in frail T2DM patients identified in the present study may lead to an impairment in liver function, illustrated by the altered serum Alb concentration and transaminase activities.

As discussed above, BM < 53 kg (*p* = 0.012) was also identified to be an independent risk factor for frailty in the present study, and as the severity of frailty worsened (for CFS grades 1 to 7), BM decreased. Usually, adipose tissue and fat deposition are necessary for growth in adolescence [33, 34]. However, in middle age, metabolic syndrome is characterized by visceral fat deposition and an inverse relationship between DHEA-S concentration and BMI [35]. An anti-obesity effect of DHEA has been shown in both humans and animals [31, 36, 37], which involves the inhibition of 11β-hydroxysteroid dehydrogenase type 1 [38] and the activation of dual-specificity protein phosphatase [39], a target gene of DHEA [40]. Although the apparently paradoxical effect of DHEA in middle-aged and elderly people remain to be explained, DHEA-S is thought to play a role in maintaining homeostasis by controlling fat deposition, but in a manner that is dependent on life stage.

One limitation of the present study was that we could not evaluate sarcopenia in all of the participants. However, in the 108 participants (32 frail and 76 non-frail) in whom this was possible, those who were frail showed a significantly higher prevalence of sarcopenia than those who were not. This finding is consistent with the concept that sarcopenia is an essential factor determining frailty [8].

## Conclusions

Hypoalbuminemia, low AST activity, and low BM were found to be independent risk factors for frailty in elderly T2DM patients, strongly suggesting relative malnutrition in this group. In addition, DHEA-S may be important for the maintenance of liver function and BM. The reduction in DHEA-S and the increase in the cortisol/DHEAS ratio may be involved in the mechanism of the effects of malnutrition in elderly T2DM patients.

## Data Availability

The datasets used and/or analyzed during the current study are available from the corresponding author on reasonable request.

## Abbreviations

CFS: The Clinical Frailty Scale
T2DM: Type 2 diabetes
HDL-C: High-density lipoprotein-cholesterol
SBP: Systolic blood pressure
RBC: Red blood cell count
Hb: Hemoglobin concentration
AST: Aspartate aminotransferase
ALT: Alanine aminotransferase
LDL-C: Low-density lipoprotein-cholesterol
eGFR: estimated glomerular filtration rate
BM: Body mass
BMI: Body mass index
DBP: Diastolic blood pressure
EWGSOP: European Working Group on Sarcopenia in Older People
ORs: Odds ratios
ROC: Receiver operating characteristic
Alb: Albumin
NAFLD: Non-alcoholic fatty liver disease
